# Treatment of anomalous arterial supply to the basal segment of left lung with aortic stent graft

**DOI:** 10.1016/j.jvscit.2022.05.010

**Published:** 2022-07-03

**Authors:** Keita Yano, Kayo Sakon, Atsushi Takamori, Narihisa Yamada, Masato Sasaki, Takaaki Koshiji

**Affiliations:** a Department of Cardiovascular Surgery, Faculty of Medical Sciences, University of Fukui, Fukui, Japan; b Department of Thoracic Surgery, Faculty of Medical Sciences, University of Fukui, Fukui, Japan

**Keywords:** Anomalous lung vessel, Lobectomy, Thoracic endovascular aortic repair

## Abstract

The patient was a 53-year-old man with a history of recurrent sputum. An anomalous systemic arterial supply to the basal segment of the left lung with an aneurysm of the aberrant artery detected on three-dimensional computed tomography angiography. Before left lower pulmonary lobectomy and aberrant artery resection, thoracic endovascular aortic repair was performed to block the blood flow to the aberrant artery aneurysm. Prior blockade of the blood flow to the aneurysm minimized the risk of aneurysm rupture and bleeding during lobectomy, yielding a good postoperative outcome.

An anomalous systemic arterial supply to the basal segment of the left lung is a rare congenital abnormality in which the normal pulmonary arteries are missing, although the pulmonary basal segment will show normal bronchial drainage. Abnormal arteries will flow from the aorta to replace the missing pulmonary arteries and eventually return to the pulmonary veins. This disease causes bloody sputum and can lead to aneurysms and rupture of the abnormal arteries. The present patient provided written informed consent for the report of his case details and imaging studies.

## Case report

**Fig 1:**
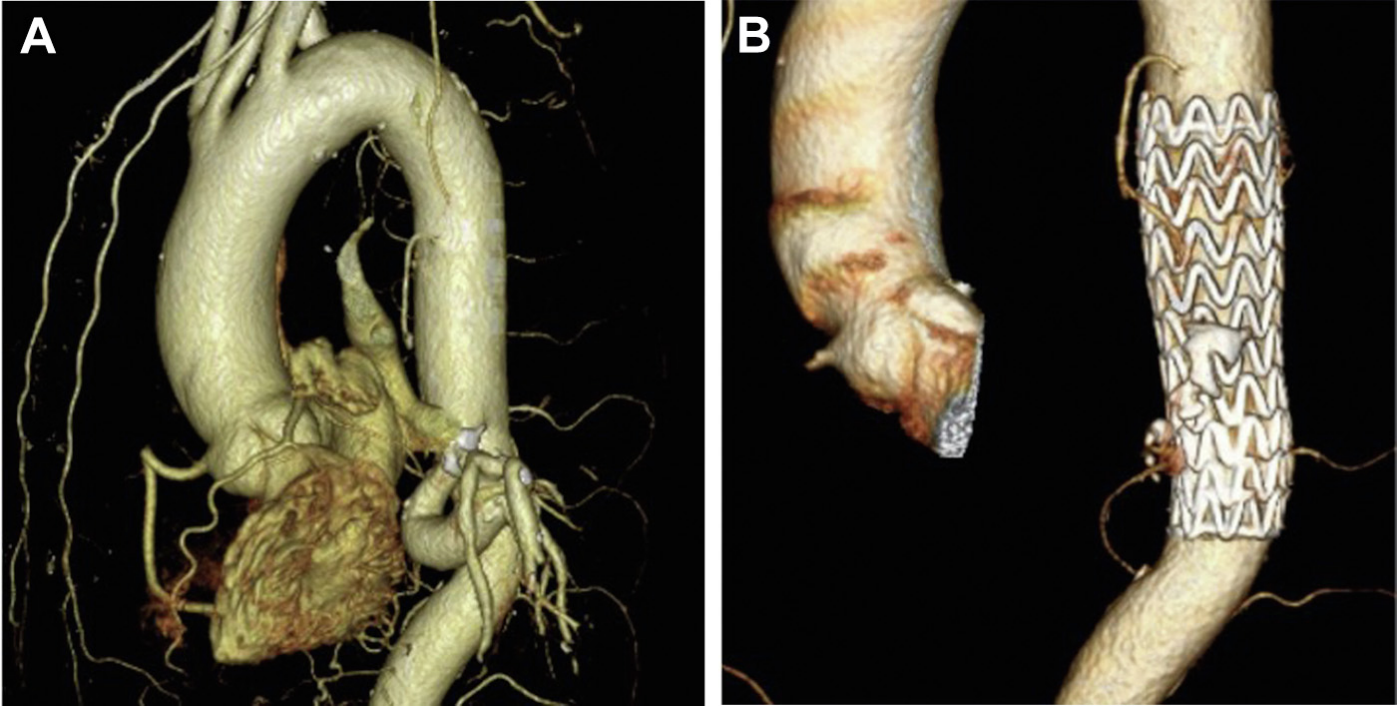
**A,** Preoperative contrast-enhanced computed tomography image. An enlarged aberrant artery bifurcating from the descending aorta directly into the left lung base area can be seen. **B,** Contrast-enhanced computed tomography image after thoracic endovascular aortic repair. The aberrant artery can no longer be seen.

The patient was a 53-year-old man with bloody sputum. All vital signs were normal, and he did not have anemia. Three-dimensional computed tomography angiography (3D-CTA) at our hospital showed an aberrant artery from the aorta to the basal segment of the left lung with noticeable dilatation and meandering, with some areas enlarged to as much as 21 mm (Fig 1, *A*). No obvious abnormality was observed on chest radiography. A left lower lobectomy and resection of the aberrant artery were considered necessary. However, because the aberrant artery was aneurysmal, the risks of bleeding and rupture were considered high owing to intraoperative passivity of the lower lobe of the lung and vessel dissection. We, therefore, decided to first perform thoracic aortic stent graft insertion to block antegrade blood flow to the aberrant artery, followed by left lower lobe pulmonary resection and abnormal artery resection. A thoracic aortic stent graft was inserted through the right femoral artery with the patient under general anesthesia. A 10-cm stent graft (cTAG; W.L. Gore & Associates, Newark, DE) was placed around the lower end of T8, where the aberrant artery had bifurcated. Because the descending thoracic aorta, peripheral to the bifurcation of the aberrant artery, was 5 mm smaller in diameter than the central side, a tapered stent graft (31-mm diameter on the central side and 26-mm diameter on the peripheral side) was used. The postoperative 3D-CTA showed no antegrade blood flow to the aberrant artery (Fig 1, *B*).

**Fig 2:**
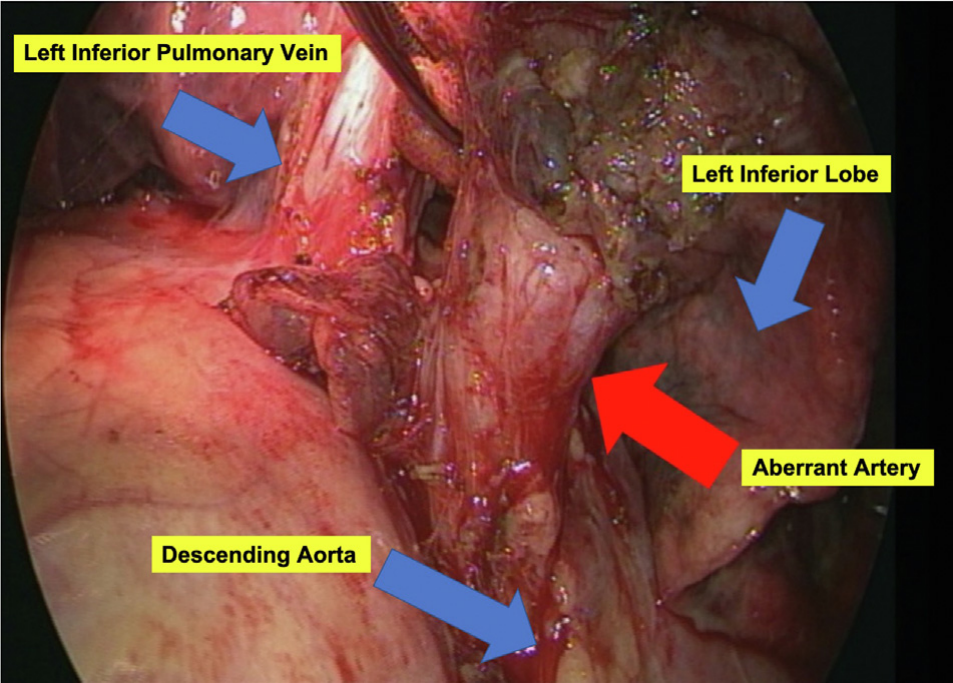
Intraoperative image of left lower lobectomy and resection of the aberrant artery. No surrounding adhesions were observed, and the aberrant artery was easily dissected and exposed (*red arrow*). No arterial pulsation from the aberrant artery was present.

**Fig 3:**
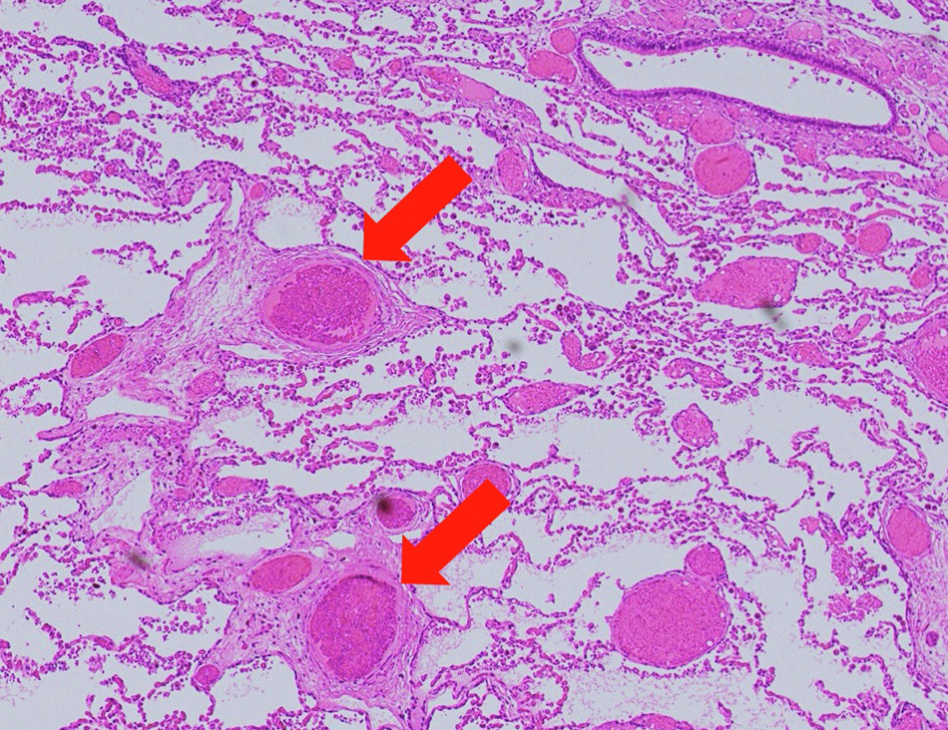
Pathologic specimen from the removed lower lobe of the left lung. Hematoxylin-eosin staining was performed (original magnification ×40). Aberrant arterial growth and dilatation can be seen (*red arrow*).

A left lower lobectomy and abnormal arterial resection were performed 1 month after thoracic endovascular aortic repair. The reason for waiting 1 month for lobectomy was that thrombus formation of the aberrant arteries and pulmonary vessels was expected. Furthermore, because the lungs have blood flow from the bronchial arteries, they will not become necrotic and have no risk of infection or pain. The surgery was performed with the patient under general anesthesia with thoracoscopic assistance by placing a 12-cm anterolateral incision in the sixth intercostal space around the midaxillary line and inserting a trocar into the eighth intercostal space. No intrathoracic adhesions were found, and the aneurysmal aberrant artery showed no pulsations and was safely resected using a surgical stapler (Fig 2). The amount of intraoperative bleeding was minimal, and no blood transfusions were required. No postoperative complications developed, and the patient was discharged on postoperative day 6. Postoperative 3D-CTA did not show any aberrant arteries. On postoperative histopathologic examination, cholesterol crystals and granulation tissue were seen within the wall of the abnormally branched basilar pulmonary artery, in addition to vascular growth and vasodilation into the lung parenchyma (Fig 3).

## Discussion

In the past, this disease was considered Pryce type 1 intralobar pulmonary sequestration.^1^ However, because the bronchial branches will be normal and no sequestered lung will be present, Painter et al^2^ proposed anomalous systemic arterialization of the lung without sequestration. This disease can be diagnosed by confirming the presence of abnormal blood vessels flowing into the lungs from the aorta, pulmonary artery inflow defects in the basal segment of the lung, and normal bronchial branching. The symptoms can include bloody sputum and dyspnea, but most patients will remain asymptomatic.^3^ This disease can be a risk factor for pulmonary hypertension and heart failure owing to the presence of left–left shunting,^3^ and death from massive hemoptysis has been reported.^4^ Therefore, surgery should be considered necessary. In addition, reports have described good outcomes with embolization of the abnormal vessel alone. However, when the abnormal vessel has developed aneurysms, the risk exists for difficult embolization and rupture during treatment.^5^^,^^6^

Two strategies for surgical resection must be discussed. First, methods of aberrant artery treatment can include direct ligation and dissection, embolization with coils or plugs, and blockage of blood flow to the aberrant artery with stent grafts. Scattered reports have described each of these options. Kestenholz et al^7^ reported 14 patients who had undergone lung resection using video-assisted thoracic surgery (VATS) with direct dissection of the aberrant artery using a stapling device, 1 of whom had undergone open conversion because of bleeding from the aberrant artery. Direct surgical dissection can damage the artery, which is likely to be vulnerable owing to chronic inflammation, and can lead to massive bleeding.^8^ We believe stent grafting can be an effective bailout procedure for bleeding. However, a sheath, wire, and aortic occlusion balloon must be inserted through the left brachial artery before VATS. If it is inserted through the femoral artery, migration can occur easily owing to the development of hypertension after aortic occlusion. The most important point is to perform the operation in a hybrid operating room. Pulmonary wedge resection after embolization with coils or plugs has also been reported.^8^ However, the concern with this approach is that embolization of the distal part of the feeding artery can lead to the formation of collateral blood channels and incomplete blockage of peripheral blood flow, with subsequent recurrence of the symptoms and infection. In addition, embolic materials can migrate to the periphery, leading to embolization of nontarget arteries.^8^ In addition, the largest size of the Amplatzer vascular plug (AGA Medical, Plymouth, MN) used for embolization is 22 mm. Thus, other methods should be considered for cases in which the aberrant artery aneurysm has increased to a diameter greater than what the plug can accommodate. One complication of thoracic aortic stent grafting that requires attention is paraplegia. Thus, this method should be selected carefully in accordance with the indications. Regarding the reimbursement prices for each medical device in Japan, the surgical staplers used for direct dissection cost ∼$300 each and coils and plugs costs ∼$1400 for a plug, $600 for a detachable coil, and $100 for a push-able coil. Finally, thoracic stent grafts cost >$1000 per graft, clearly making the use of stent grafts more expensive.

Second, the method of thoracotomy should be discussed. If safety can be guaranteed, performing the procedure with less invasive methods would be desirable. In addition, segmentectomy require cutting between the zones of the lung carries a high risk of bleeding, because determining the growth of the aberrant arteries can be difficult. Therefore, with sufficient respiratory reserve, we believe lobectomy will be safer. To further ensure safety, prior treatment of aberrant arteries can be considered useful. In the present patient, an aberrant artery aneurysm was identified. Thus, with consideration of the risks of rupture and migration after embolization with coils or plugs, it decided to perform lobectomy with VATS and ensure safety by blocking the blood flow to the aberrant artery with prior thoracic aortic stent grafting. However, stent grafting carries a risk of paraplegia and is more expensive than the other treatment methods. The choice of treatment method should be determined after considering the advantages and disadvantages of each option.
